# A Narrative Review of the Complex Relationship between Pregnancy and Eye Changes

**DOI:** 10.3390/diagnostics11081329

**Published:** 2021-07-23

**Authors:** Nicoleta Anton, Bogdan Doroftei, Ovidiu-Dumitru Ilie, Roxana-Elena Ciuntu, Camelia Margareta Bogdănici, Ionela Nechita-Dumitriu

**Affiliations:** 1 Department of Ophthalmology, Faculty of Medicine, University of Medicine and Pharmacy “Grigore T. Popa”, University Street, No. 16, 700115 Iasi, Romania; anton.nicoleta1@umfiasi.ro (N.A.); Roxana-elena.ciuntu@umfiasi.ro (R.-E.C.); camelia.bogdanici@umfiasi.ro (C.M.B.); dumitriuionela@yahoo.com (I.N.-D.); 2 Ophthalmology Clinic, “Saint Spiridon” Emergency Clinic Hospital, Independence Avenue, No. 1, 700111 Iasi, Romania; 3 Department of Mother and Child Medicine, Faculty of Medicine, University of Medicine and Pharmacy “Grigore T. Popa”, University Street, No. 16, 700115 Iasi, Romania; 4 Origyn Fertility Center, Palace Street, No. 3C, 700032 Iasi, Romania; 5 Department of Biology, Faculty of Biology, “Alexandru Ioan Cuza” University, Carol I Avenue, No. 20A, 700505 Iasi, Romania; ovidiuilie90@yahoo.com

**Keywords:** pregnancy, glaucoma, neuro-ophthalmology, diabetes, intraocular pressure, diabetic retinopathy

## Abstract

Pregnancy is a condition often characterized by changes that occur in different parts of the body. Generally, the eyes suffer several changes during pregnancy that are usually transient but may become permanent at times. This may occur due to the release of placental hormones and those of maternal endocrine glands and fetal adrenal glands. Due to hormonal influences, physiological ocular changes during pregnancy have been shown in Caucasian women, so corneal sensitivity, refractive status, intraocular pressure, and visual acuity may change during pregnancy. Within this review, all studies that referred to physiological aspects and to changes of ocular pathology of pregnancy, the effect of the pregnancy on pre-existing (diabetic retinopathy, neuro-ophthalmic disorders) eye disorders, postpartum ocular changes, the intraocular pressure and the effect of hypotensive ophthalmic medicine during pregnancy, the connection between pregnancy and the neuro-ophthalmic pathology, as well as the role of anesthesia were analyzed.

## 1. Introduction

Pregnancy is a complex physiological process that affects all organic systems. Eye changes during pregnancy can be physiological or pathological. Physiological changes can involve any of the body’s organs, including the eye and visual system [1,2,3]. The effect of pregnancy on the eyeball involves a broad spectrum of physiological and pathological changes [1,2,3]. Corneal sensitivity, refractive status, intraocular pressure, and visual acuity may change during pregnancy [4,5,6]. Pathological changes are divided as follows: eye changes that occur for the first time during pregnancy (keratoconus, dry eye syndrome, etc.), pre-existing ocular pathologies but modified by pregnancy (glaucoma, diabetic retinopathy, neuro-ophthalmic pathology), and ocular manifestations of systemic diseases (pregnancy-specific diseases: preeclampsia/eclampsia/Sheehan’s syndrome) or diseases that occur more frequently during pregnancy: idiopathic intracranial hypertension and disseminated intravascular coagulation [3].

According to several studies, changes can occur as a result of the release of placental hormones, maternal endocrine glands, and fetal adrenal glands. Although most eye complications of pregnancy are mild, transient, and do not require treatment, some are occasionally severe, permanent, and require prompt referral to an ophthalmologist. In addition, some ocular complications that occur during pregnancy can provide a direct perspective on the pathophysiology of many systemic diseases [3].

The frequency of glaucoma during pregnancy seems to increase among women. There is a tendency for intraocular pressure (IOP) to decrease during pregnancy, especially in the second and third trimesters. In addition, a reduced diurnal variation in intraocular pressure and an increase in retrobulbar blood flow during pregnancy have been reported [4]. Studies show that progesterone is inversely related to intraocular pressure, with intraocular pressure decreasing significantly as pregnancy progresses [4].

Human chorionic gonadotropin (HCG) has been associated with decreased intraocular pressure in postmenopausal women [5]. In their study, Kitzmiller et al. [6] recommend the consensus panel for the medical care of pregnant women with pre-existing diabetes. They claim that an inadequate glycemic monitoring before pregnancy and an increased value of glycosylated hemoglobin in the early stages of pregnancy are associated with the progression of diabetic retinopathy during pregnancy, with the rate of progression of diabetic retinopathy being twice as high in pregnant women as in the control group [6].

Another challenge is managing the pregnant woman with a neuro-ophthalmic disorder. Physiological changes in pregnancy make vascular disorders more common, including retinal artery occlusion, spontaneous orbital hemorrhage, and pituitary apoplexy. The treatment of neuro-ophthalmic complications of pregnancy requires an understanding of the risks of drugs [3]. Therefore, it is significant to be aware of the physiological changes, as well as the potential effects on pre-existing diseases and complications, in order to counsel women who are currently pregnant.

Thus, the present narrative review aims to offer an insight regarding all eye changes in pregnancy.

## 2. Methodology

This manuscript was conducted by respecting the procedures described previously by Green and co-authors [7].

### 2.1. Database Searches

All studies between 2000 and 2020 were included by searching the following databases: PubMed/Medline, ResearchGate, GoogleScholar, DovePress, ScienceDirect, Elsevier, and Cochrane Database of Systematic Reviews (CDSR). The search strategy included keywords to find suitable materials: “pregnancy and glaucoma”, “pregnancy and diabetes”, “pregnancy and neuro-ophthalmology”, “hormonal changes during pregnancy”, “eye disease in pregnancy”, and “visual acuity in pregnancy”.

The adopted PubMed string was (pregnancy[Title/Abstract] AND glaucoma[Title/Abstract]) AND diabetes[Title/Abstract] AND neuro-ophthalmology[Title/Abstract] AND hormonal changes[Title/Abstract] AND eye disease[Title/Abstract] AND visual acuity[Title/Abstract].

### 2.2. Eligibility Criteria

Studies reported in English and in other languages, case reports, reviews, and animal studies not older than 2000 were selected. Conference posters, computational simulations, and Letters to the Editor were excluded.

### 2.3. Study Selection

We identified a total of 159 articles (158 articles performed on human patients, 1 on rabbits).

## 3. Results

### 3.1. Ocular Anterior Segment Changes in Pregnancy

During pregnancy, changes are observed in the anterior segment: eyelids, lacrimal apparatus, and cornea. At the level of the eyelids, a brown pigmentation is observed around the eyes (pregnancy chloasma) secondary to the increase of melanogenesis and melanocytosis in pregnancy [3].

The dry eye syndrome secondary to the direct effects on the lacrimal acinar cells of prolactin, transforming growth factor beta 1 (TGF-β1), and epidermal growth factor is found at the level of the lacrimal apparatus [3,5]. Skare et al. [8] identified an increased incidence of dry eye syndrome among pregnant women compared to the control group.

Additionally, corneal thickness, curvature, and biomechanical parameters of the cornea have been shown to be affected by variations in sex hormones. Corneal thickness increases due to water retention in pregnancy, as well as its radius of curvature that accompanies a change in contact lens tolerance [3]. In a study on 60 pregnant women, Goldich et al. [9] found changes in corneal curvature explained by estrogen and progesterone, as well as relaxin (increased during pregnancy) that degrades collagen and matrix metalloproteinases, thus weakening the corneal collagen matrix. The authors did not find any difference between the central corneal thickness in pregnant women compared to the control group and between the CH and CRF values in the Ocular Response Analyzer (ORA). It is recommended that a new optical correction (glasses or lenses) be prescribed to pregnant women a few weeks postpartum [3]. Cases of keratoconus have been reported in pregnant women. Soeters et al. published two clinical cases of two pregnant women who developed keratoconus at the second pregnancy under risk factors (myopia, previously wearing contact lens) and under the influence of estrogen and cortisol. Both patients reported decreased visual acuity, case one at the end of the second pregnancy, and the second postpartum case at the second pregnancy [9,10,11,12]. At the level of the lens, transient impairment of the accommodation was observed, and when it is shown, it is recommended to postpone the refractive surgery until a stable refraction is achieved [3].

### 3.2. Pregnancy-Associated Hormonal Changes with Ophthalmic Effects

Progesterone has an antagonizing effect on the effects of endogenous corticosteroids in the trabecular meshwork. This is manifested by facilitating the elimination of aqueous humor, but with normal production and decreased intraocular pressure [13,14]. We can state that the progesterone is inversely proportional to the value of intraocular pressure [15].

Human chorionic gonadotropin (HCG) is associated with the decrease of intraocular pressure in postmenopausal women [13]. Similar results were observed in an experimental study performed in rabbits (normal or ovariectomy) after intravenous or intravitreal administration of HCG. At the ocular level, HCG stimulates the formation of cyclic adenosine monophosphate (cAMP), which results in decreased production of aqueous humor [14]. Another physiologically present hormone in pregnancy, relaxin, is associated with decreased IOP by increasing the discharge of aqueous humor. However, its intramuscular injection resulted in decreased IOP in both men and women with glaucoma [15].

### 3.3. Glaucoma and Pregnancy

The frequency of glaucoma during pregnancy seems to increase among women, as some women wait longer to get pregnant. Therefore, we need to improve our understanding of glaucoma management in this very challenging population.

Intraocular pressure and central corneal thickness are significantly higher in the first trimester compared to the second and third trimesters of pregnancy, respectively, with postpartum values (Table 1 and Table 2) [3,9,16,17,18,19,20]. There are cases when intraocular pressure increases and is difficult to manage [21,22]. Nitric oxide (NO), entothelin 1 (ET-1), and eicosanoids have a vasodilating effect and, implicitly, facilitate the drainage of aqueous humor. Ding et al. studied the role of human chorionic gonadotropin (HCG) in the dynamics of intraocular pressure in laboratory animals. They observed that HCG administration is associated with decreased intraocular pressure [5]. Additionally, the intraocular pressure at multiparous is lower than the possible primiparous secondary to anxiety, stress, or insomnia that determines the predominant action of the sympathetic nervous system (SNS) [2].

Studies have also shown that intraocular pressure is lower in a sitting position compared to supination or lateral decubitus [17]. Minimal glaucoma therapy is not associated with increased intraocular pressure or changes in visual field, but if pressure is increased and perimeter change progress, additional medication is required [23,24]. It should be noted that all these pressure variations and the success of glaucoma treatment in pregnancy largely depend on the associated systemic pathologies [25].

#### 3.3.1. Medical Treatment of Glaucoma

According to the Food and Drug Administration (FDA) classification (Table 3), there are no ocular hypotensive drugs in class A drugs that can be safely administered during pregnancy. Class B contains only brimonidine and dipivephrine. All other classes of ocular hypotensive drugs have caused side effects in animal studies and are therefore not administered to the pregnant patient (beta blockers, local and systemic carbonic anhydrase inhibitors, prostaglandin analogues, parasympathomimetics). Classes D and X include drugs with a proven harmful effect. Thus, no antiglaucoma drug falls into this category [4,23,25].

Table 4 summarizes the drug classes and their effects on pregnancies according to the latest studies in the literature. Beta blockers cross the placenta and cause bradycardia and fetal arrhythmia; newborns exposed to this drug require careful cardiovascular surveillance in the first 24–48 h postpartum [24,25,27,28,29]. Brimonidine should be discontinued near birth because it crosses the blood–brain barrier (BBB) and causes central nervous system (CNS) depression and apnea in children [25,28,30,31]. Topical dorzolamide used in the treatment of glaucoma in pregnancy as a third line of treatment was not associated with an adverse effect [4,22]. The American Academy of Pediatrics has approved the use of carbonic anhydrase inhibitors during lactation with the recommendation of careful monitoring by oral administrators [25]. Cholinergic agents (parasympathomimetics) are rarely used, being poorly tolerated by young patients, including pregnant women [23,24,25]. Prostaglandin analogues belong to FDA class C [28]. They cross the placenta and cause uterine contractions that can lead to spontaneous abortion [32], premature birth (PTB), or low birth weight (LBW), so most ophthalmologists do not recommend them [33].

Selective laser trabeculoplasty is effective both before and during pregnancy [37] when the morphology of the chamber angle allows [28,38]. Success is compromised in chamber angle abnormalities (Axenfeld–Rieger syndrome or aniridia) [28,38].

Selective argon laser trabeculoplasty is less effective in young patients (under 50) [28,38]. Numerous successful cases of laser trabeculoplasty in pregnant women are cited [22,39,40].

Laser peripheral irritodomy or laser iridoplasty is a safe treatment and can be performed as a prophylaxis of acute closure of the chamber angle in pregnant women without real effects on the fetus [41].

Cyclophotocoagulation is performed under intracameral, suportonian, or retrobulbar anesthesia as an alternative to classical incisional surgery [41]. In some cases, micropulse and cyclophotocoagulation laser therapy can be performed [41]. A case when laser cyclophotocoagulation with retrobulbar anesthesia was performed in a pregnant woman at the beginning of pregnancy recorded as successful in the control of postoperative pressure [21] is cited in the literature. Shunt implantation in pregnant patients can be performed safely for pressure control that cannot be controlled with local treatment or laser therapy [4]. Recent studies show success in IOP control secondary to Ahmed or Baerveldt valve implantation [4], but these devices show an evolving hypertensive phase [42,43]. Additionally, trabeculectomy can be performed with an ExPress Ologen Colagen Matrix (biodegradable) mini-shunt implant [44,45,46,47] or minimally invasive glaucoma surgery with XEN gel stent implantation without antimetabolites [48].

#### 3.3.2. Surgical Treatment (Trabeculectomy)

It is an exceptionally rare alternative when the disease progresses and IOP is not adequately controlled with medication and laser [41]. It presents many challenges and exposes both the pregnant woman and the fetus to significant vital risks if performed under general anesthesia [4]. Peribulbar or southenone anesthesia with lidocaine is a safer alternative [4]. Surgery in the second and third trimesters may be accompanied by profound hypotension in the supine position by compression of the aorta and vena cava by the uterus [22,34,49], and the patient’s left lateral decubitus is recommended to avoid these complications [4]. Postoperatively, erythromycin (FDA class B) is administered both during pregnancy and lactation [28,50]. Trabeculectomy is an alternative for pregnant women in need of this intervention [41,51], which has been successful in decreasing intraocular pressure [21]. The use of antimetabolites (mitomycin C and 5-fluorouracil) is prohibited in pregnant patients [41]. According to the FDA, these two substances are in class X, having teratogenic effects well documented in animal studies [52,53]. In pregnancy, the serum level of placental growth factor (PGF) [54] as a ligand for the endothelial growth factor receptor (VEGF) has high values, and this increases the risk of filter failure in trabeculectomy because the “wound” heals at an increased rate compared to normal [4].

Table 5 shows the therapeutic plan adopted for three pregnant women with high ocular blood pressure values under maximum antiglaucoma treatment. Control of intraocular pressure was successful in all three cases, and the newborns did not have any health problems [4].

#### 3.3.3. Anesthesia during Pregnancy

The use of anesthetics in pregnant women is performed only when absolutely necessary and in the lowest amount and concentration adapted to the interventions performed [4]. Most local anesthetics used (lidocaine, prilocaine, and etidocaine are class B drugs according to the FDA) had no teratogenic effects. Bupivacaine and mepivacaine can cause fetal bradycardia. These are drugs that belong to the FDA class C in animal studies an can cause teratogenic effects [4]. In a study, 11–23% of pregnant women were exposed to prenatal local anesthetics without an increase in the incidence of fetal malformations [4,22]. There are few studies in the literature on the use of anesthetics in ophthalmology [4,25]. Studies show that anterior local or southerly anesthesia is better tolerated than retrobulbar or systemic anesthesia in terms of the risk of systemic absorption and the occurrence of side effects [4,28].

#### 3.3.4. Glaucoma Treatment in Pregnancy

The progression of glaucoma during pregnancy can vary between individuals, so medical or surgical ophthalmic treatment should be done depending on the period of preconception, but especially in the first trimesters of pregnancy, because some antiglaucoma drugs can cause fetal side effects until spontaneous abortion. The treatment plan must take into account the trimester of pregnancy and the complications that may occur; the table below describes the drugs and related studies that can be administered during preconception; trimesters I, II, and III; birth; and postpartum for a good pressure release without harmful effects on the fetus (Table 6).

### 3.4. Pregnancy and Neuro-Ophthalmological Pathology

Managing a pregnant woman with a neuro-ophthalmic disorder can be a challenge. Physiological changes in pregnancy make vascular diseases more common, including retinal artery occlusion, spontaneous orbital hemorrhage, and pituitary apoplexy. In the third trimester of pregnancy there is an asymptomatic increase in sensitivity to contrast in the visual field, which is a completely reversible change postpartum [3]. During pregnancy, blood volume and cardiac output are increased by 30–50%, and the increase in extracellular fluid (approximately 2 L at term) causes an increase in serum osmolarity [63,64,65]. According to NMR measurements, the volume of the ventricles of the brain decreases in pregnancy and expands. These changes happen secondary to alkalosis, and postpartum hormones influence the brain, returning it to its initial dimensions [63,64,65]. The level of prolactin is increased in the cerebrospinal fluid. This hormone plays a role in attachment and breastfeeding [66].

The pituitary gland increases by 30%, and the volume doubles, returning to normal at 1–2 weeks postpartum. Rarely, physiological changes of the pituitary gland encountered in pregnancy cause chiasmatic compression [67].

Orbital varicose veins begin with a feeling of fullness around the eyes. Orbital ultrasound, CT, or MRI is recommended. Varicose veins can become symptomatic due to increased blood volume. Little is known about their treatment [68]. Complications are similar to those found in pregnant women: bleeding, glaucoma, and thrombosis. Cesarean delivery is not mandatory but can be performed with epidural anesthesia, “rest and descent”, and vacuum or forceps [68].

Spontaneous orbital bleeding may occur in the first quarter (additional to nausea and vomiting) or during pregnancy (additional to Valsalva maneuver) [68]. The symptoms are sudden onset diplopia, proptosis, and pain [69]. Anticoagulant treatment is a risk factor. The diagnosis is orbital ultrasound, CT, or MRI. The bleeding usually stops spontaneously [68].

Stroke is more common postpartum. Cerebral vascular diseases represent 0.47–6% of the causes of maternal mortality [70]. Cardiac embolism (endocarditis, paradoxical embolism) is the most common cause of acute brain phenomena [68]. Peripartum cardiomyopathy, of unknown etiology and incompletely misunderstood pathophysiology, may present as acute heart failure that is usually not reversible, with patients showing symptoms a few weeks or months postpartum [71]. Amniotic fluid embolism is a rare but very severe complication that occurs with multiple arterial occlusions in both the brain and eye [72].

Papilledema in pregnancy raises several problems of diagnosis and treatment and is a challenge for the ophthalmologist [73]. It may be unilateral (differential diagnosis with ischemic, compressive, inflammatory optic neuropathy) or bilateral [74]. It can manifest as a rapidly progressive optic neuropathy secondary to a hormone-sensitive orbital apex schwannoma or optic neuritis secondary to optic neuromyelitis during pregnancy, which exacerbates optic neuromyelitis [75]. Thus, any pregnant woman with papillary edema should be monitored for blood pressure and proteinuria to rule out malignant hypertension or pregnancy toxemia. It is also recommended that an MRI be performed to rule out space replacement formations, obstructive hydrocephalus, venous hypertension caused by arterio-venous malformation, or venous thrombosis. If systolic blood pressure and MRI are normal, CSF pressure at lumbar puncture opening and CSF examination are measured. Causes of papillary edema secondary to CSF changes include aseptic (sarcoidosis), infectious (viral, bacterial, fungal, tuberculosis), and tumor (lymphoma, leukemia) causes. If this investigation is also negative, the patient is included in the category of intracranial hypertension (uremia, tetracycline derivatives, excess vitamin A (prenatal screening is recommended for hypervitaminosis A [76]), sleep apnea, steroidal anti-inflammatory drugs) with or without identifying the etiological factor [76]. Significant recent weight gain is another risk factor [68,73,74,76]. The causes of papilledema in a pregnant woman are similar to those in the nonpregnant patient, with the exception of the additional possibility of preeclampsia/eclampsia. Two conditions, idiopathic intracranial hypertension (IIH) and cerebral venous thrombosis (CVT), are more frequent in pregnant patients than in the nonpregnant general population. The pregnant female with papilledema should have her blood pressure taken to determine if preeclampsia is a possibility, and once ruled out, a magnetic resonance imaging examination should be performed without contrast (after 18 weeks) followed by cerebral spinal fluid analysis if not contraindicated [68].

Increased intracranial pressure may be primary (idiopathic) or secondary (cerebral vein thrombosis, meningitis, eclampsia, space replacement formation). Cerebral venous thrombosis is one of the most common causes [68,77]. The symptoms may be confused with those of intracranial hypertension [68,77]. Convulsions or hemiplegia may occur [68,77]. The possible causes may include the V Leiden factor (it reaches values of 8 or higher during pregnancy), deficiency of protein C or S, prothrombin gene, homocysteinemia, and autoimmune pathologies (systemic lupus erythematosus and anticardiolipin antibodies [68,77]. Although thrombosis usually occurs postpartum, there are situations in which it can be triggered during childbirth [68,77]. Treatment is significant, and it has been shown that women who received heparin had a 50% lower risk of death than those without anticoagulant treatment without any peri- or postnatal complications [68,78]. CSF drainage through lumbo-peritoneal or ventriculo-peritoneal shunt can be challenging [79,80]. Treating headache in pregnancy is another challenge [68,78].

Regarding neuro-ophthalmic impairment in severe preeclampsia and eclampsia, it has been observed that hypertension is an important risk factor for maternal mortality according to various studies [81,82]. Eclampsia and preeclampsia are two entities characterized by severe hypertension and proteinuria that begin at more than 20 weeks of gestation [74]. From an ophthalmological point of view, the patient can complain of impaired chromatic sense, headache, blurred vision, and bright flashes [68]. Cases of Purtscher-like retinopathy associated with serous retinal detachment in a pregnant woman with preeclampsia have been reported [82] but also secondary to HELLP syndrome [83].

In terms of cranial neuropathies, the most common is damage to the VII cranial nerve (facial nerve), namely Bell’s palsy [10]. The incidence of Bell’s palsy is increased in the third trimester [84,85]. The etiology of Bell’s palsy is not fully understood [84]. Trochlear nerve palsy can be found in the third trimester of pregnancy, with excellent postpartum recovery or loss of total or partial accommodation during pregnancy or childbirth. These changes occur secondary to the growth of interstitial fluid around the nerve, causing compression. In general, the prognosis is good, as prednisone treatment is not necessary [68].

### 3.5. Pregnancy and Diabetic Retinopathy

Inadequate glycemic control before pregnancy and increased glycosylated hemoglobin in the early stages of pregnancy are associated with the progression of diabetic retinopathy during pregnancy [86,87,88,89] in both type 1 and type 2 diabetes [90,91]. Progression of diabetic retinopathy is associated with hypertension, diabetic nephropathy, and preeclampsia [6]. The progression of diabetic retinopathy is associated with hypertension, diabetic nephropathy, and preeclampsia [86,90,91,92,93,94,95]. It has been shown that the treatment of hypertension with angiotensin receptor blockers is associated with a decrease in the rate of progression of retinopathy. Even in normotensive patients but with diabetes, the same treatment mentioned above can stop or even involve the evolution of diabetic retinopathy [86,96,97,98]. The presence of macular edema in early pregnancy in pregnant women with type 1 diabetes is associated with severe progression of diabetic retinopathy [95]. The risk of developing diabetic retinopathy in pregnant women with type 2 diabetes is lower than in the case of type 1 diabetes [33,87,91]. A glycosylated hemoglobin value of 5.6% was observed to be associated with a low rate of progression of diabetic retinopathy for both type 1 and type 2 diabetes [99]. The rapid decrease in blood glucose through intensive treatment at any stage of evolution is also associated with the rapid progression of ocular changes [86,88,90,91]. However, the longer the duration of diabetes and the longer the insulin requirement, the higher the risk of their retinal changes [86,91,93,100,101,102]. Until now, it has not been shown that there is a link between the rate of progression of diabetic retinopathy and the age of pregnant women [99,103]. In these cases, frequent ophthalmological examination is recommended [99]. Ophthalmic screening of pregnant women with diabetes has the same recommendations in both types of diabetes [91,99]. It is recommended that women who have diabetes and want a child have proper glycemic control before becoming pregnant [88,99]. There are various studies in the literature that detail the therapeutic behavior of pregnant women with diabetes, and it is important to study them carefully to make the best decision for the patient [104,105,106,107,108,109,110].

### 3.6. Pregnancy and Other Retinal Changes

Retinal vascular occlusions may occur secondary to disseminated intravascular coagulation [111] secondary to HELLP syndrome, V Leiden factor mutation, and thrombophilia [112,113,114]. Hereditary or acquired thrombophilia is associated with an increased risk of maternal thrombosis but also with a risk of miscarriage or eclampsia. Kurtz et al. argue that this diagnosis should be seriously considered if the patient has a history of spontaneously stopped pregnancies in order to administer appropriate treatment and to reduce the risk of both the mother and the course of the pregnancy [115]. Most studies claim that thrombophilia is manifested by retinal venous occlusions, especially in patients under 50 [116,117,118], and this is especially true for people who also have the following risk factors: high blood pressure, diabetes, and glaucoma [115,116,119,120,121,122,123]. When retinal venous occlusions cannot be explained by the presence of carotid disorders [124], antiphospholipid antibody syndrome [125], or Behcet’s disease [126,127] it is important to consider hereditary thrombophilia and hypofibrinolysis [119,120,121,122,123,125,128,129].

Cases of patients who complained of fleeting amaurosis, non-arteritic ischemic optic neuropathy, or retinal arterial or venous occlusions, and who were subsequently diagnosed with hereditary thrombophilia, are cited in the literature [116,119,120,121]. The diagnosis and prompt treatment of thrombophilia is not only relevant from an ophthalmological point of view but also prevents maternal thrombosis and the risk of spontaneous abortion [130,131,132,133,134]. Choroidal neovascularization may be secondary to myopia [135,136] or idiopathic [137]. Pregnancy is a risk factor for central serous chorioretinopathy [138,139]. Increased intrathoracic or intra-abdominal pressure may occur in the eye through Valsalva retinopathy [140,141,142]. Pregnancy-induced hypertension is associated with multiple ocular changes of practical relevance. The following were observed: conjunctival vascular abnormalities, hypertensive retinopathy, exudative retinal detachment, vitreous or preretinal hemorrhage ischemic optic neuropathy, and hypertensive choroidopathy [143] Changes in the optic or retinal nerve are associated with low birth weight [143,144] and are closely related to low APGAR score. Multiple studies suggest that assessing the fundus of pregnant women in this situation is vital to prevent fetal side effects [143,144].

### 3.7. Myopia and Pregnancy

Most studies claim that during the second and third trimesters, there was a significant increase in the corneal curvature that remits completely with the birth and cessation of breastfeeding. After the end of the lactation period, there is a change in contact lens tolerance, which is perfectly normal, to that from before pregnancy [145]. It is recommended that a new optical correction (glasses or lenses) be prescribed to pregnant women a few weeks postpartum [3]. Another study suggests that changes in visual acuity during pregnancy, especially from the first trimester, can be caused by changes in corneal thickness that are thought to be due to hormonal changes during this period. Study participants resumed normal visual acuity after birth. Hormonal changes may also play a role in progestins and estrogens, which increase the permeability of the lens to water, thus reducing the refractive index [146]. Myopia is associated with increased frequency of retinal degenerative changes, which are the risk factors of intra- and postpartal ophthalmological complications. Wielgos et al. analyzed the degenerative lesions detected in ophthalmological examination (including peripheral retinal lesions) as potential risk factors for status of the eyes in terms of delivery in myopic women. Degenerative retinal lesions are present in one fourth of pregnant women. Both the severity and type of the lesions are not associated with severity of myopia. They concluded that among pregnant patients, retinal lesions occur in patients with more advanced maternal age. Thus, it should be considered that ophthalmological examination remains an important prophylactic modality in retinal disorders, especially in primary retinal detachment due degenerative disorders [147].

## 4. Conclusions

Analyzing the above data, we can say that it is always vital to look at the pregnant woman as a complex patient, as a whole, and any investigation or therapeutic measure must be very well documented so as not to have side effects on her or the fetus. From an ophthalmological point of view, as observed in the reported studies, eye changes either induced by the pregnancy or aggravated by it are extremely complex and sometimes difficult to manage. Knowing the different ocular changes during pregnancy helps to differentiate physiological changes from pathological eye disorders in pregnant women, to which treatments may differ. The frequency of glaucoma during pregnancy seems to increase among women. There was an increased incidence of dry eye syndrome in pregnant women, secondary to the direct effects on the lacrimal acinar cells of prolactin, TGF beta 1, and epidermal growth factor.

Given the lack of reports on the management of pregnant patient glaucoma and the impossibility of conducting clinical trials in this group of patients, there are no guidelines for the management of these clinical situations. Intraocular pressure decreases significantly as the pregnancy progresses, while the level of intraocular pressure is closely related to the level of progesterone, being inversely proportional. Future studies should highlight the role of placental chorionic gonadotropin (HCG) on the dynamics of intraocular pressure. Additionally, another approach would be the role of relaxin, another physiologically present hormone in pregnancy, which would be associated with a decrease in intraocular pressure by increasing the discharge of aqueous humor. With all the information indicating that intraocular presence usually decreases during pregnancy, many glaucoma patients continue to need medical and surgical treatment, and glaucoma may progress. Thus, choosing the type of antiglaucoma medication according to the FDA classification is vital.

The progression of ophthalmic diseases such as keratoconus and diabetic retinopathy in pregnancy is another direction of studies to be followed.

Another key aspect is the management of the pregnant woman with a neuro-ophthalmic disorder that can be a challenge for the ophthalmologist. The treatment of neuro-ophthalmic complications of pregnancy requires an understanding of the risks of drugs. Taking optimal care of the mother will usually lead to the best care for the baby.

The relationship between the obstetrician and the ophthalmologist must be permanently open during pregnancy. Periodic ophthalmologic evaluation could detect and treat possible ocular changes early, and the quality of life and visual prognosis of patients should be positive both in the long and short term.

## Figures and Tables

**Table 1:** Variation of intraocular pressure during the three trimesters of pregnancy and postpartum.

No. of Patients	Median Age	PIO Trim I	PIO 2nd Trim	PIO 3rd Trim	PIO Postpartum	Reference
25	29 ± 3	13.81 ± 2.08	12.96 ± 1.9	12.42 ± 2.08	13.31 ± 2.07	[17]
54	27.37 ± 5.64	-	-	13.39 ± 2.93	15.35 ± 2.76	[18]
117	27.5	14.7 ± 2.2	13.2 ± 2.0	11.0 ± 1.3	14.2 ± 1.8	[19]

**Table 2:** Central corneal thickness values during the third trimester and postpartum.

No. of Patients	Median Age	CCT 3rd Trim	Postpartum CCT	Reference
25	29 ± 3	573.68 ± 24.03	562.49 ± 23.40	[17]
54	27.37 ± 5.64	539.85 ± 33.38	535.69 ± 34.95	[18]

**Table 3:** Pregnancy antiglaucoma medication according to FDA classification [26].

	Pregnancy antiglaucoma medication according to FDA classification
Class A	-
Class B	Brimonidine
Class C	Beta blockers Prostaglandins Carbonic anhydrase inhibitors Pilocarpine
Class D	-
Class X	-

**Table 4:** Antiglaucoma medication and side effects.

Drug Class	FDA Class	Features	Fetal Side Effects	Cautions	Lactation Administration	Reference
Beta blockers	C	Cross the placenta	Teratogenic risk in the first trimester Fetal bradycardia Fetal arrhythmia Timolol apnea in the newborn	Timolol—Careful cardiovascular monitoring 24–48 h newborn	Yes	[4,23,25,26,27,28,29,30,34,35]
Prostaglandin analogues	C	Cross the placenta	Uterine muscle contractions Miscarriage Premature birth Low birth weight	Low half-life. It can be given during lactation.	Yes	[4,23,29,32,33]
Brimonidine	B	It is secreted in breast milk	CNS depression Apnea in newborn	It must be stopped near birth because it crosses the BBB with CNS depression and apnea in the newborn.	No	[4,25,28]
Acetazolamide	C	-	Administered in the first trimester caused limb malformations in rats, mice and hamsters (studies before 2000) Sacrococcygeal teratoma Renal tubular acidosis	-	Yes	[4,21,25,36]

**Table 5:** Surgical management in glaucoma with success in control of intraocular pressure [4].

	Diagnostic	Treatment	Anesthesia Type
Case 1	Juvenile glaucoma	OU Trabeculectomy without antimetabolites (2nd trimester first eye, 3rd trimester second eye)	Topical anesthesia lidocaine gel and subconjunctival lidocaine
Case 2	Juvenile glaucoma	OU Ahmed valve implantation (second trimester, then third trimester) after unresponsive selective laser trabeculoplasty	Topical tetracaine and subconjunctival and sub-Tenon’s lidocaine
Case 3	Juvenile glaucoma	OU Baerveldt valve implantation (third trimester)	General anesthesia

**Table 6:** The type of drugs they can administer and related studies.

Treatment Period	Management	Reference
Before conception	- informing the patient about the risks of antiglaucoma medication and the follow-up plan. - complete ophthalmological examination. - glaucoma staging, target intraocular pressure. - if necessary, perform laser or surgical treatment before pregnancy.	[4,25,34]
First trimester	- stop antiglaucoma medication/add brimonidine/occlude tear points. - it is not recommended to initiate therapy with prostaglandin analogues. - surgery is not recommended to prevent teratogenic side effects and the risk of premature birth.	[28,32,41,55]
Second trimester	- brimonidine first line treatment. - beta blockers can be added only if they are absolutely necessary but with careful monitoring of the pregnant woman and the fetus. - prostaglandin analogues are third-line but may be associated with a risk of premature birth. - any newly recommended medication is administered only after the approval of the obstetrician and neonatologist.	[41]
Third trimester	- after 36–37 weeks of gestation, fetal exposure to various active substances is associated with apathy after birth complications. - STOP brimonidine. - beta-blockers may cause fetal arrhythmia, bradycardia, hypotension and CNS depression. - prostaglandin analogues are associated with an increased risk of premature birth. - dorzolamide severe metabolic acidosis in a newborn. - laser trabeculoplasty can be performed in all trimesters. Although less effective in controlling long-term intraocular pressure, these interventions can stabilize intraocular pressure until the end of pregnancy when other therapeutic measures can be taken.	[25,28,31,32,33,34,41,55,56,57]
Birth	- 25% of obstetricians and 3.6% of ophthalmologists recommend that the birth be performed by cesarean section in patients with glaucoma. - recommendations arose as a result of adverse effects on maternal intraocular pressure through the Valsalva maneuver during vaginal birth.	[58,59,60,61,62]
Postpartum	- brimonidine is contraindicated because it causes depression of the central nervous system. - betablockers are carefully administered to newborns with congenital heart disease. - prostaglandins have a very short half-life which makes their administration immediately after breastfeeding reduce the risk of exposure of the newborn. - administration of topical and systemic carbonic anhydrase inhibitors has not been associated with systemic side effects.	[22,41]

## Data Availability

The datasets used and analyzed during the current study are available from the corresponding author on reasonable request.
